# Automatic measurement of the evolutionary process dynamics of primary of biliary cirrhosis

**DOI:** 10.1186/1746-1596-8-S1-S16

**Published:** 2013-09-30

**Authors:** Nicola Dioguardi, Carlo Russo, Emanuela Morenghi, Barbara Franceschini, Sonia Di Biccari, Stefano Musardo, Guido Bosticco

**Affiliations:** 1 Laboratory for metric method in Medicine, Istituto Clinico Humanitas IRCCS, Rozzano, Milan, Italy; 2 Biostatistic unit, Istituto Clinico Humanitas IRCCS, Rozzano, Milan, Italy; 3 Pavia University, Italy

## Background

PBC is a chronic inflammatory autoimmune disease [1] that evolves into cirrhosis via four stages. These are determined by the successive implementation in hepatic tissue of the following events: inflammation, destruction and regeneration of biliary tissue CK7+ and fibrosis. This paper describes a method to study of the dynamics of the disease histologic behaviour [2].

## Materials and methods

We studied 58 liver biopsy samples, from the archives of Departments of Pathological Anatomy of Istituto Clinico Humanitas IRCCS, Rozzano, Milan, Italy and the Department of Gastroenterology of Milan’s Ospedale Policlinico IRCCS, of Caucasian subjects with PBC in various stages. Each procedure was performed in accordance with the guidelines of the Ethics Committees of the hospitals involved and diagnoses were defined by the expert pathologists of these same hospitals.

Three consecutive 2-3 µm thick sections were obtained from each sample: the first was used to identify inflammatory cells; the second was stained with Sirius red to visualize collagen deposits and the third was used to visualize the biliary tree with CK7 antibody.

The histological metrization method [3] was developed in the Laboratory for the Study of Metric Methods in Medicine of the Istituto Clinico Humanitas (Rozzano, Milan, Italy). The prototype of this computerized device called HM and its controlling software were entirely designed and developed in our laboratory. The Metrizer is a totally computer-driven machine that automates the focusing of the microscope and the exclusion of Glisson’s capsule from the computation of fibrotic islets. It performs reproducible metric measurements from digitalized images of the entire histological section, giving results within a few minutes. This method measures all notable liver structures bared of any property not involved in the measurement.

## Statistical analysis

The data are given as numbers and percentages or mean or median values and ranges where appropriate. All variables were log-transformed in order to approximate a Gaussian distribution and normalized to a (0,1) range to allow for comparison between variables. Thus the final data are expressed in percentage of the interval between 0 and 1. All of the analyses were performed with Stata10 [http://www.stata.com].

## Results and discussion

**Figure 1:**
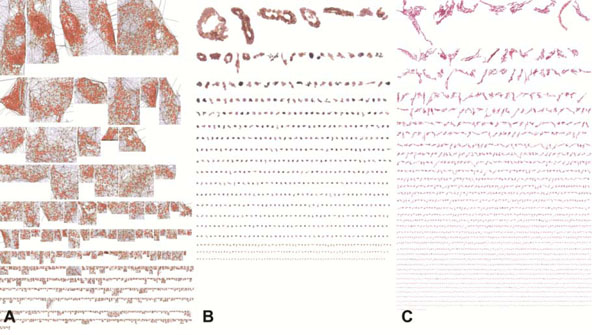
Catalogue of the multifarious fragments of liver structures measured by HM.; **A.** Inflammation cell clusters **B.** CK7; and **C.** fibrosis elements, The black image is a Glisson membrane fragment excluded by the Metrizer.

***Inflammation*** (Figure1A) was identified by defining the borders around clusters of mononuclear cells (lymphocytes) with Delaunay's triangulation method [4].

This method identifies a line that connects the centers of outermost cells that are distanced ≤20 μm. This line is arbitrarily considered the separator of the inflammatory cells within a cluster from the mononuclear cells dispersed in the surrounding hepatic tissue.

The *inflammatory basin*, which increases constantly due to the autoimmune process, is the set of areas in a section that are covered by inflammatory cell clusters. The area of the basin covered by cluster-resident cells is called the *pure inflammatory space*, which varies with the number (density) of cells present in the clusters.

The ***intra-hepatic biliary duct*** area (Figure 1B) was metrically measured. During the course of PBC, the biliary tree duct status is determined by two components: autoimmune destruction and regeneration of intrahepatic CK7+ ductular segment.

The ***collagen islets*** forming fibrosis (Figure 1-C) were measured in linear meters; the result was corrected by the fractal dimension [5] to include details of the irregularity of their shapes The fractal dimension was obtained by means of the box counting method because the objects to be measured were “truncated fractals” [6], the fractal dimension was used as a dilation factor rather than an exponent [7] Three classes of islet magnitude were arbitrarily identified: area from 10 and 10^3^ μm^2^, from 10^3^ to 10^4^ μm^2^ and over 10^4^ μm^2^.

The ***tissue disorder*** quantitative assessment was performed with a Tectonic Index (TI), which describes the loss of tissue organization or any deviation from the natural order (a high TI indicates a high degree of tissue disorder). The TI defines the organization of liver tissues and is expressed with a range of values from 0 to 1. The TI was calculated as 1 – H, where H is Hurst’s coefficient (range zero to 1) [8], defined as D_γ_ + 1 – D, where D is the fractal dimension and D_γ_ the Euclidean dimension of the observed object. So TI = 1 – H = D - D_γ_. Numerical results of all of these parameters are summarized in Table 1.

**Table 1:** Summary of all of the metric data obtained from the structural measurements and the data used for staging purposes. Minimum, median, and maximum values of tissue parameters are given in % of total histologic section area of biopsy specimen.

Tissue parameters	Minimum	Median	Maximum
***Area of the inflammatory basin***	0.026	1.005	6.444

Area covered by resident cells	0.002	0.636	5.295

***Area of intra-hepatic biliary ducts***	0.003	0.114	0.882

Small areas (10-10^3^ μm)	0	0.009	0.105

Medium-sized areas (10^3^-10^4^ μm)	0.001	0.006	0.033

Large areas (>10^4^ μm)	0	0	0

***Area of fibrosis***	0.30	1.456	24.4

Small areas (10-10^3^ μm)	0.008	0.092	0.521

Medium-sized areas (10^3^-10^4^ μm)	0.001	0.026	0.203

Large areas (>10^4^ μm)	0.01	0.075	1.005

***Neo-angiogenesis***	0.030	0.586	3.474

***Hemopoietic stem cells***	0	0.006	0?

***Tissue disorder (TI)***	0.072	0.274	0.643

In order to approach the PBC behavioural dynamics [9], the first key was to set into the interval (0,1) the logarithmic transformation and normalization of the measures of CK7+; tissue and fibrosis, taken as the most representative structural elements with which to metrically construct the liver state portrait that defines the grading and staging of the semiquantitative subjective methodologies.

A second key is the transformation of portrait scalars into a single vector to reduce the multiplicity of elements of the liver section into a dot-like geometrical figure. This translation into vectorial arithmetic in classic dynamics is crucial for the construction of the dot-like geometrical operator, called dynamic *particola* that represents the whole system.

**Figure 2:**
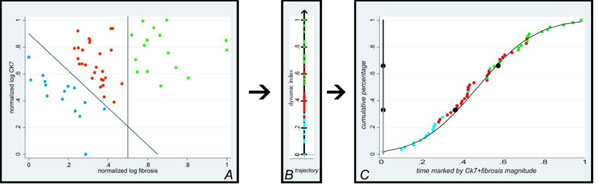
A): The set of dynamic *particulae* resembling a Gibbs cloud-point obtained expressing as vector magnitudes the normalised log value of CK7+ areas and the normalised log value of fibrosis in our patients. B) The points are transferred to the oriented line of real numbers representing the standardised trajectory of the process. C) An ogival cumulative curve orders the dynamic *particula* within the three clouds in panel A. This curve is sub-divided into three tertiles marked by blue points and represents the trajectory of the overall dynamic process of PBC from α to ω.

In order to gain a method to study the system’s behaviour dynamics [9], the immune CK7+ biliary ductules resulting from the destruction-regeneration and intrahepatic fibrosis, considered a set of phenomena that generates the irreversible tectonic disorder leading to cirrhosis. The values of vectors representing irreversible parameters, resulting from the HM measurement, were taken to create the *particola*. The vectorial transformation is obtained by plotting the value of CK7 area on the *y* and the value of fibrosis areas on the *x* orthogonal axes. The value of the modulus of this resultant sum in the orthogonal space is taken as the Newtonian dot-like dynamic *particola* that shows an instant of the PBC behaviour (Figure 2A). The set of points (each a *particola*) on the x-y orthogonal space, resembles a Gibbs cloud of points, each expressing in log scale the magnitude of the vectorial value of a *particola* with a scalar. These scalars ordered on a real number line, the simplest state space, will graphically show the trajectory that is the reference of the dynamic behavior of PBC process. (Figure 2B).

Furthermore, we plotted the value of each particula on the y axis (ranging from 0 to 1) versus the disease timing, identified with all particulae, on the x axis (Figure 2C). Next, we identified the tertiles on this curve that discriminate three phases. This term used in our new method is different from the ‘stages’ characterising semiquantitative static descriptions. As a result, the three phases are extrapolated by increasing amounts of particolae formed by fibrosis and CK7+ tissue. In particular, phases 1, 2, and 3 represent early, intermediate, and final disease to feature mean particula values of 0.2075, 0.4722, and 0.7386, respectively.

The inflammation was excluded from the constitutive components of the trajectory describing the course of PBC process, for its reversibility. This different behaviour is due to the entropy produced in the interior of the inflammation process that is not transferred across its boundaries into the surrounding environment [10].

## Conclusions

The method we constructed, with its technology, strongly reduced the computational time and improved the liver tissue structure recognition and description. As these tools allowed this first study of PBC behaviour in terms of physics of dynamics, this paper supports the hypothesis that the long periods of cessation in the history of dynamic knowledge were due to the very higher rapid theoretic development toward facilitating computation devices [11,12]. The technology introduced into our methodology facilitated the study of PBC behaviour by the following points:

1) Exclusion of the inflammatory infiltrates from dynamic study, as they do not produce topic stable entropy deposits. Collagen interstitial deposition generating fibrosis is hepato-cellular necrosis dependent.

2) Standardization of a strictly objective evaluation producing scalars by metrizing the images of the histological structures of the liver section.

3) Metrical measurement also of the smallest dispersed islets of fibrosis, and tiny CK7+ biliary ducts normally undetected with optical microscope.

4) Transformation of scalars into vectors leading to vectorial PBC histological section portraits. A homogeneity was created with this operation that maintains the concepts of mixture and identity of the mixed elements in the sum of CK7+ biliary ducts and fibrosis vectors that define the *particola*, geometric figure which graphically will trace the process trajectory.

5) Description of PBC evolution by the cumulative curve of the *particolae*, each representing an actual state of the processes and taking this planar curve as the ideal finite trajectory α–ω) of entire PBC process.

6) Definition of past and future percentage of the disease course with its *particola* on the trajectory.

7) The score of PBC evolution into three phases on its trajectory and describe the ideal course of the process based on mathematical measurements.

To conclude let us say that any correlation is recognizable between the metrical data of our case-list, divided into three phases by the rules of dynamics and the semi-quantitative data, divided into four stages according to the rules of the method of Scheuer. (Table 2) For example the 20 specimen classified in our higher phase III included 9 patients (45%) classified by the semi-quantitative Scheuer classification at minor levels of staging. Where is the truth?

**Table 2:** Distribution in the three stadia of the dynamic trajectory of the patients’list classified according to Scheuer’s classification.

		Scheuer classification			
**Phase**		**1**	**2**	**3**	**4**

**I**	**19 (33%)**	11 (58%)	7 (37%)	1 (5%)	0

**II**	**19 (33%)**	11 (58%)	1 (5%)	6 (32%)	1 (5%)

**III**	**20 (34%)**	9 (45%)	2 (10%)	5 (25%)	4 (20%)

## List of abbreviations

PBC: Primary biliary cirrhosis; IRCCS: Institute for Scientific Research Hospitalisation and Care; HM: Histologic Metrizer; TI: Tectonic Index

## Competing interests

No conflicts of interest exists.

## Authors’ contributions

ND wrote the manuscript and ideated the theory around the machine and dynamics and the machine itself, CR ideated the software and constructed the machine, EM did the statistical analysis and contributed to revision of the text, BF, SDB and SM did the histological preparations, GB revised the text.
